# Supplementary material for: Deletion of wfs1 Impairs Oligodendrocyte Precursor Cells Dorsal Distribution and Myelination Through the wfs1-hmgcs1 Axis in Zebrafish
Source: Biology (Basel). 2026 Aug 16;15(16):1402. doi: 10.3390/biology15161402 (PMC13510151; doi:10.3390/biology15161402)
Supplement: Supplementary file 1 [file biology-15-01402-s001.zip › File S1-Transcriptome sequencing data and differentially expressed genes (DEGs) in wfs1-- zebrafish at 5 dpf/2.KEGG/wfs1KOvsWT/ALL/src/dre00900.html]

dre00900


- dre:559054

- Up regulated genes

559054\_1(2.91197574019232)

- Down regulated genes

559054(-1.4113026040815)

- dre:100003383

- Up regulated genes

100003383(3.39697101268788)

- Down regulated genes

100003383\_1(-2.23974139439836)

- dre:557720

- Up regulated genes

557720\_1(5.16286229747943)

- dre:334380

- Up regulated genes

334380\_1(4.57377854253414)

- Down regulated genes

334380(-2.10881202849194)

Close
